# Synthesis and crystal structure of tetra­methyl (*E*)-4,4′-(ethene-1,2-di­yl)bis­(5-nitro­benzene-1,2-di­carboxyl­ate)

**DOI:** 10.1107/S2056989024002676

**Published:** 2024-03-28

**Authors:** Artjom Businski, Thuy C. Ta, Niklas Gindullis, Christian Näther, Rainer Herges

**Affiliations:** a Otto-Diels-Institut für Organische Chemie, Universität Kiel, Otto-Hahn-Platz 4, D-24098 Kiel, Germany; b Institut für Organische Chemie, Leibniz Universität Hannover, Schneiderberg 1 B, D-30167 Hannover, Germany; c Institut für Anorganische Chemie, Universität Kiel, Max-Eyth-Str. 2, D-24118 Kiel, Germany; Vienna University of Technology, Austria

**Keywords:** crystal structure, synthesis, stilbene derivative, diazo­cine

## Abstract

The title compound, C_22_H_18_N_2_O_12_, was obtained as a by-product during the planned synthesis of 1,2-bis­(2-nitro-4,5-dimethyl phthalate)ethane by oxidative dimerization starting from dimethyl-4-methyl-5-nitro phthalate. To identify this compound unambiguously, a single-crystal structure analysis was performed. The asymmetric unit consists of half a mol­ecule that is located at a centre of inversion. As a result of symmetry restrictions, the mol­ecule shows an *E* configuration around the double bond. Both phenyl rings are coplanar, whereas the nitro and the two methyl ester groups are rotated out of the ring plane by 32.6 (1), 56.5 (2) and 49.5 (2)°, respectively. In the crystal, mol­ecules are connected into chains extending parallel to the *a* axis by pairs of C—H⋯O hydrogen bonds that are connected into a tri-periodic network by additional C—H⋯O hydrogen-bonding inter­actions.

## Abstract 2

In the crystal structure of the title compound the two phenyl rings are coplanar, whereas the nitro and the two methyl ester groups are rotated out of the ring plane. The mol­ecules are linked by inter­molecular C—H⋯O hydrogen bonding into a tri-periodic network.

## 1. Chemical context

**Figure 1:**
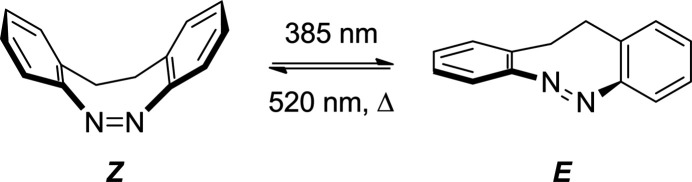
Light-induced reversible isomerization between the thermodynamically stable *Z* and the metastable *E* isomer of the parent diazo­cine with different wavelengths in the visible range. In addition, thermal relaxation leads to re-isomerization.

In recent years, mol­ecular photoswitches have gained much attraction because of their wide range of potential applications, *e.g*. as photoresponsive materials (Pang *et al.*, 2019 ▸) or as drugs (Kobauri *et al.*, 2023 ▸). Bridged azo­benzenes, so-called diazo­cines, are photoswitches, in which the thermodynamically stable *Z* isomer can be reversibly converted to the metastable *E* isomer through irradiation with visible light of different wavelengths (Fig. 1 ▸). Compared to azo­benzenes, these compounds exhibit superior photophysical properties such as well-separated absorption bands, high quantum yields and high switching efficiencies (Siewertsen *et al.*, 2009 ▸). Additionally, the light-driven *E*/*Z* isomerization leads to a reversible mol­ecular movement between the bent, sterically demanding *Z*, and the stretched *E* isomer (Moormann *et al.*, 2019 ▸), which can be used for reversible expansion and contraction between polymer strands (Burk *et al.*, 2023 ▸) or reversible receptor–substrate binding (Cabré *et al.*, 2019 ▸; Ewert *et al.*, 2022 ▸).

The general synthesis of diazo­cines usually includes two key reactions: the formation of the ethyl­ene unit and the azo group. Common synthesis strategies for C—C linkage include an oxidative dimerization (Moormann *et al.*, 2017 ▸), a Sonogashira cross-coupling (Maier *et al.*, 2019 ▸), Wittig reaction (Samanta *et al.*, 2012 ▸) or organolithium-mediated reductive couplings (Li *et al.*, 2020 ▸). In contrast, N—N formation is usually achieved by reductive/oxidative coupling starting from di­nitro/di­amino compounds (Moormann *et al.*, 2017 ▸; Maier *et al.*, 2019 ▸; Klockmann *et al.*, 2021 ▸) or by a Cu-catalysed cascade reaction using diiodide compounds (Li *et al.*, 2020 ▸). Unfortunately, late-stage functionalization after formation of the diazo­cine ring is difficult. Therefore, substituents have to be introduced at an earlier stage in synthesis, ideally before the oxidative C—C bond-formation stage.

Along these lines, we aimed at the synthesis of a tetra­methyl­ester substituted diazo­cine with two ester groups each in the *meta* and *para* positions to the azo group. After ester hydrolysis, the carb­oxy­lic acids were converted to the cyclic anhydrides, which were reacted with different amines to yield the corresponding imides. The tetra­ester, therefore, is an ideal precursor for further functionalization of the diazo­cine chromophore. 
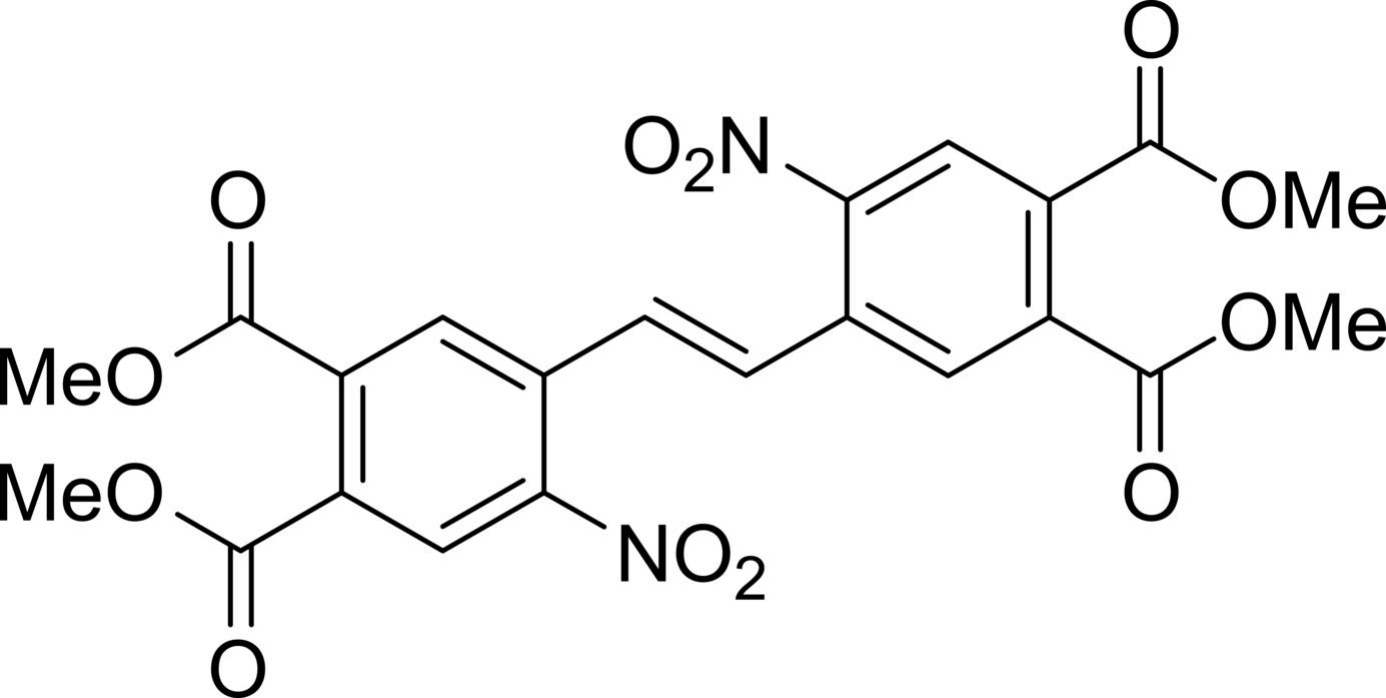


**Figure 2:**
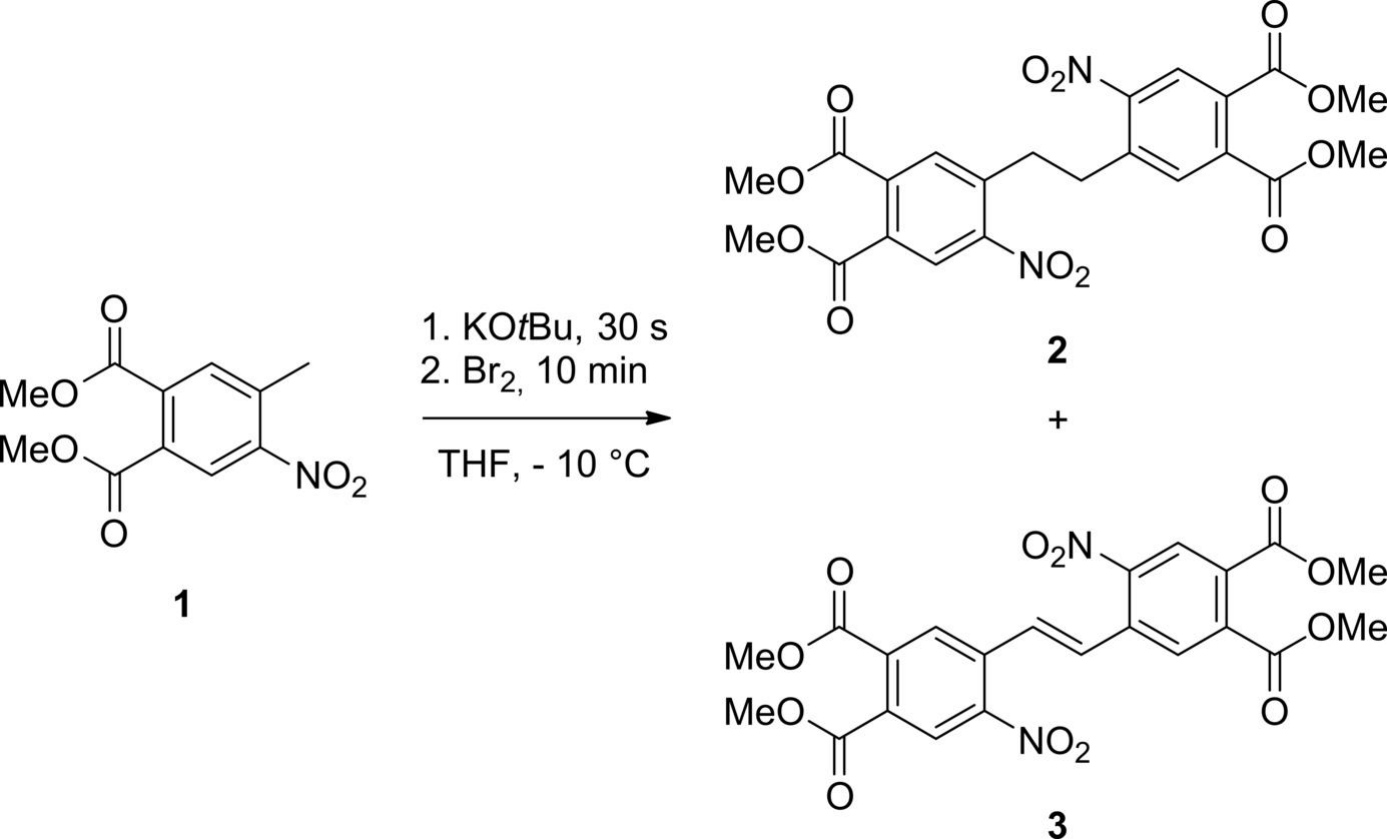
Reaction scheme to obtain the title compound (**3**) as a by-product.

Starting from commercially available 4-methyl­phthalic anhydride, we carried out nitration and esterification reactions according to literature procedures (Hao *et al.*, 2019 ▸) yielding dimethyl-4-methyl-5-nitro phthalate (**1**, Fig. 2 ▸). Dimerization of **1** by oxidative C—C bond formation was achieved through consecutive addition of potassium *tert*-butoxide and bromine in tetra­hydro­furan yielding a crude product. According to ^1^H NMR spectroscopy, the raw material contained a structurally similar by-product in addition to the expected main product 1,2-bis­(2-nitro-4,5-dimethyl phthalate)ethane (**2**, Fig. 2 ▸). From vapour diffusion experiments of the crude product, we obtained crystals of the pure product, which were characterized by single crystal structure analysis, proving that (*E*)-1,2-bis­(2-nitro-4,5-dimethyl phthalate)ethene, C_22_H_18_N_2_O_12_, (**3**) has formed as by-product (Fig. 2 ▸).

## 2. Structural commentary

**Figure 3:**
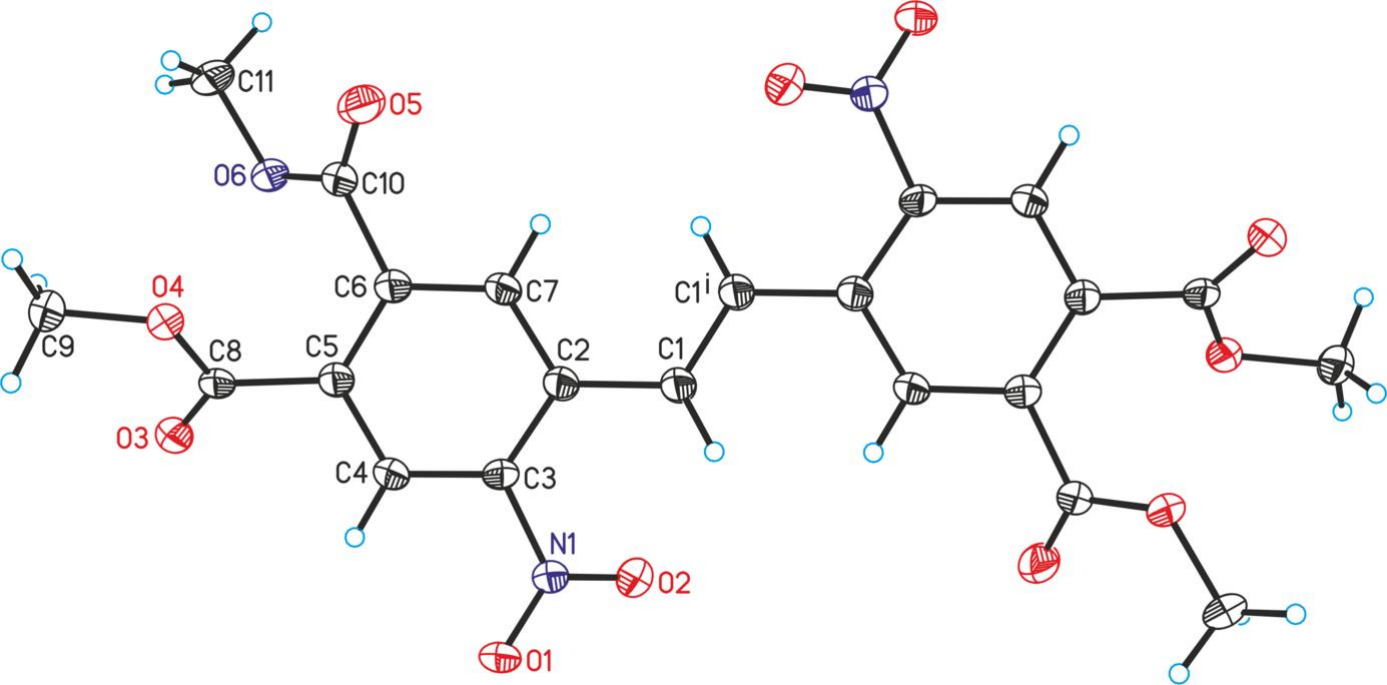
Crystal structure of the title compound with labelling and displacement ellipsoids drawn at the 50% probability level. [Symmetry code: (i) −*x*, −*y* + 1, −*z* + 1.]

**Figure 4:**
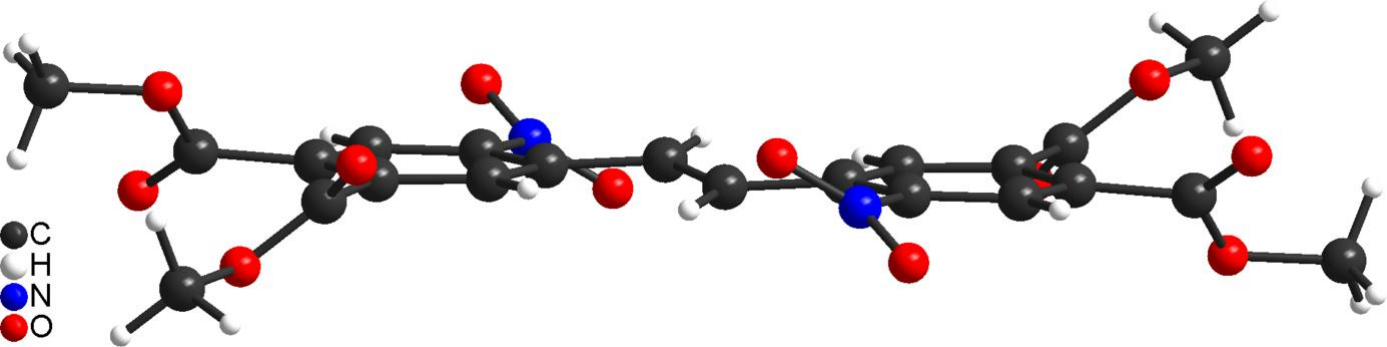
Side view of the title compound showing the torsion of the nitro and the ester groups.

The asymmetric unit of **3** consists of half of a mol­ecule that is located at a centre of inversion (Fig. 3 ▸). As a result of symmetry restrictions, the mol­ecule shows the *E* configuration around the double bond, which can be traced back to steric hindrance. Both phenyl rings are oriented in a coplanar fashion (Fig. 4 ▸). The nitro group is rotated out of the phenyl ring plane by 32.6 (1)°, whereas the dihedral angles between the six-membered ring and the two methyl ester groups amount to 56.5 (2) and 49.5 (2)°, respectively.

## 3. Supra­molecular features

**Figure 5:**
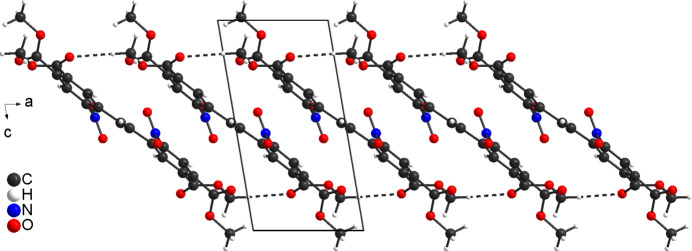
Crystal structure of the title compound along the *b* axis in a view of the hydrogen-bonded chains. Inter­molecular C—H⋯O hydrogen bonding is shown as dashed lines.

**Figure 6:**
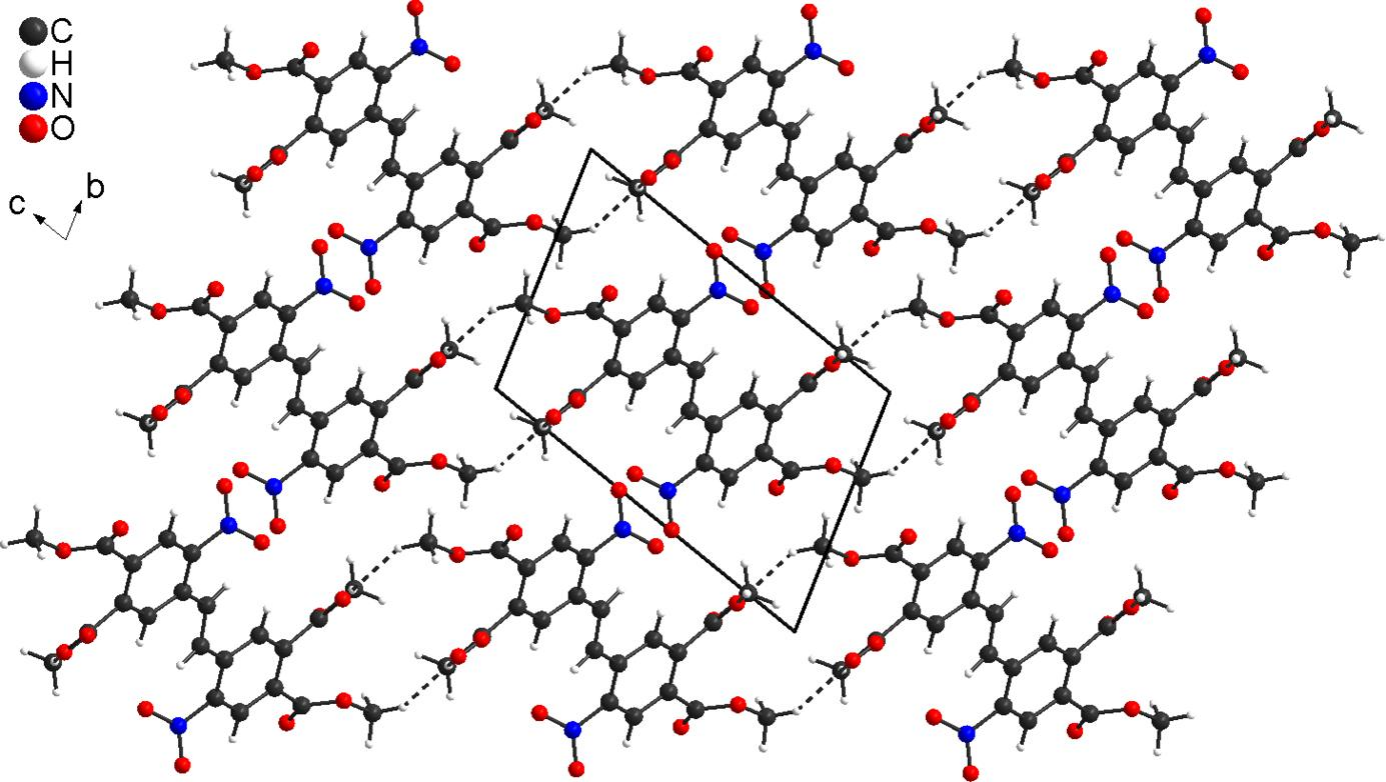
Crystal structure of the title compound in a view along the *a* axis. Inter­molecular C—H⋯O hydrogen bonding is shown as dashed lines.

**Figure 7:**
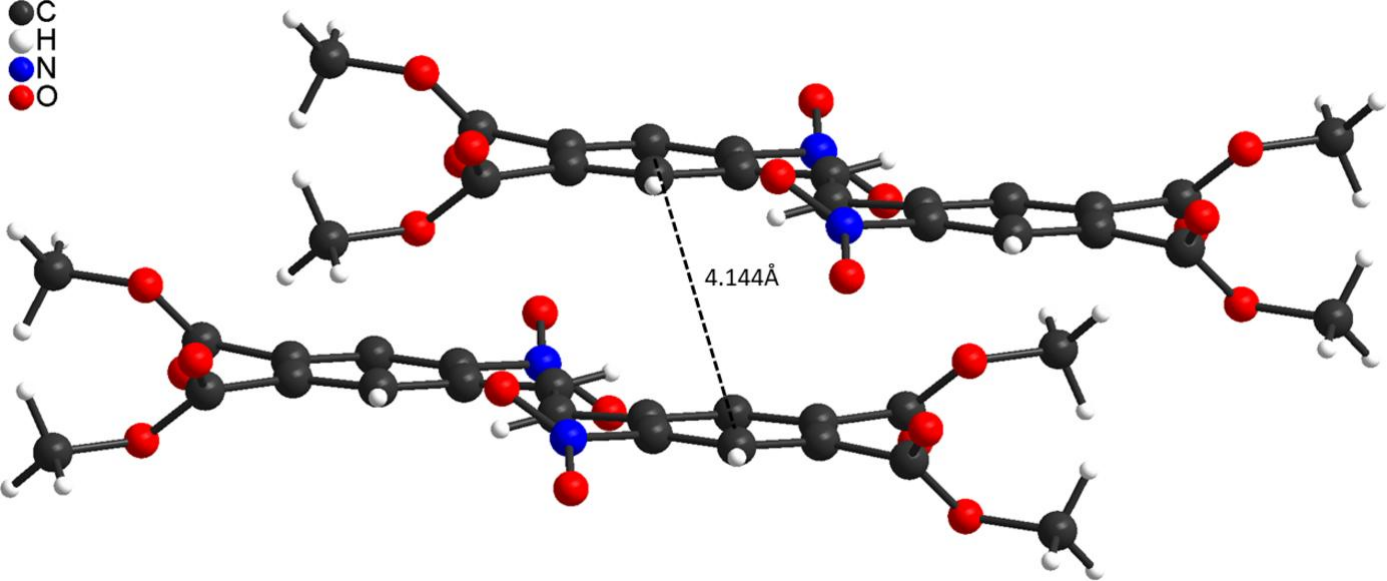
View of two neighbouring mol­ecules. The distance between the centroids of the six-membered rings is given.

In the crystal of **3**, the mol­ecules are connected into chains by centrosymmetric pairs of C—H⋯O hydrogen bonds between the methyl hydrogen atom H11*C* and the carbonyl oxygen atom O5 (Fig. 5 ▸). The C—H⋯O angle is close to linearity, indicating that this is a significant inter­action (Table 1 ▸). These chains propagate parallel to the *a* axis, with each chain surrounded by six neighbouring chains (Fig. 6 ▸). The chains are additionally linked into a tri-periodic network by centrosymmetric pairs of C—H⋯O hydrogen bonds between the methyl hydrogen atom H9*B* and the carbonyl O atoms O5, forming 16-membered rings that are located around centres of inversion (Fig. 6 ▸). The corresponding O⋯H distance and the C–H⋯O angle point to a weaker inter­action (Table 1 ▸). There is one additional C—H⋯O hydrogen bond but with a significant longer O⋯H distances (Table 1 ▸), which consolidates the packing. Finally, the mol­ecules are arranged in a way that phenyl rings of neighbouring mol­ecules are parallel but the ring planes are shifted relative to each other and the distance between the centroids of the six-membered rings amount to 4.144 (1) Å, which does not point to significant π–π inter­actions (Fig. 7 ▸).

## 4. Database survey

A search of the CSD (version 5.43, last update March 2023, Groom *et al.*, 2016 ▸) using CONQUEST (Bruno *et al.*, 2002 ▸) revealed that thousands of stilbene derivatives are reported. With only nitro groups in an *ortho*-position, only three hits are found, including *trans*-1,1′-(ethene-1,2-di­yl)-bis­(2-nitro­benzene [1,2-bis­(2-nitro­phen­yl)ethene] or *trans*-2,2′-di­nitro­stilben (refcodes WIXJIZ and WIXJIZ01, Bulatov & Haukka, 2019 ▸; Blelloch *et al.*, 2021 ▸). In addition, a hydrate of *trans*-1,1′-(ethene-1,2-di­yl)-bis­(4-carboxyl­ato-2-nitro­benzene) (refcode JAWYIS, Song *et al.*, 2017 ▸) matches the search criterion. Finally, there is one zinc carboxyl­ate compound with carboxyl­ate groups in the 4-position (refcode BOZYOG, Li *et al.* 2014 ▸). With each two carboxyl­ate or ester groups in *ortho* positions to each other, no hits are found. In fact, there is no compound reported in the CCDC that is more closely related to the title compound.

## 5. Synthesis and crystallization

**General**

Dimethyl-4-methyl-5-nitro phthalate (**1**) was prepared according to the literature (Hao *et al.*, 2019 ▸) starting from 4-methyl­phthalic anhydride (> 98%), which was purchased from TCI. Potassium *tert*-butoxide (> 97%) was purchased from TCI and bromine (99%) from Thermo Scientific. Tetra­hydro­furan (99.9%) was purchased from Fisher Scientific and dried using the solvent purification system PureSolv MD 5 from Inert Corporation.

**Synthesis**

Under a nitro­gen atmosphere, dimethyl-4-methyl-5-nitro phthalate (**1**, 10.0 g, 39.5 mmol) was dissolved in dry tetra­hydro­furan (330 ml) and cooled to 263 K. Potassium *tert*-butoxide (5.76 g, 51.3 mmol) was added in one portion. The reaction mixture was stirred for 30 s, whereupon bromine (2.02 ml, 39.5 mmol) was immediately added. After complete addition, the reaction mixture was stirred at 263 K for 10 min and then quenched with ice. The precipitate was filtered off, washed with the smallest possible amount of ice-cold ethyl acetate and dried *in vacuo*. The crude product was obtained as a pale-yellow solid.

**Crystallization**

Single crystals of **3** were obtained by vapour diffusion using chloro­form/methanol as solvent/anti­solvent.

## 6. Refinement

Crystal data, data collection and structure refinement details are summarized in Table 2 ▸. C-bound hydrogen atoms were positioned with idealized geometry (methyl H atoms allowed to rotate but not to tip) and were refined isotropically with *U*_ĩso_(H) = 1.2 *U*_eq_(C) (1.5 for methyl hydrogen atoms) using a riding model.

## Supplementary Material

Crystal structure: contains datablock(s) I. DOI: 10.1107/S2056989024002676/wm5712sup1.cif

Structure factors: contains datablock(s) I. DOI: 10.1107/S2056989024002676/wm5712Isup2.hkl

Supporting information file. DOI: 10.1107/S2056989024002676/wm5712Isup3.cml

CCDC reference: 2342598

Additional supporting information:  crystallographic information; 3D view; checkCIF report

## Figures and Tables

**Table 1:** Hydrogen-bond geometry (Å, °)

*D*—H⋯*A*	*D*—H	H⋯*A*	*D*⋯*A*	*D*—H⋯*A*
C9—H9*B*⋯O5^i^	0.98	2.42	3.308 (2)	150
C11—H11*A*⋯O3^ii^	0.98	2.52	3.3650 (19)	144
C11—H11*C*⋯O5^iii^	0.98	2.39	3.364 (2)	173

Symmetry codes: (i) 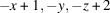 ; (ii) 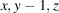 ; (iii) 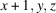 .

**Table 2:** Experimental details

Crystal data	
Chemical formula	C_22_H_18_N_2_O_12_
*M*_r_	502.38
Crystal system, space group	Triclinic, *P* 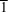
Temperature (K)	100
*a*, *b*, *c* (Å)	5.9454 (2), 7.9543 (3), 12.0673 (4)
α, β, γ (°)	72.124 (3), 79.661 (3), 86.052 (3)
*V* (Å^3^)	534.25 (3)
*Z*	1
Radiation type	Cu *K*α
μ (mm^−1^)	1.12
Crystal size (mm)	0.19 × 0.08 × 0.02

Data collection	
Diffractometer	XtaLAB Synergy, Dualflex, HyPix
Absorption correction	Multi-scan (*CrysAlis PRO*; Rigaku OD, 2022 ▸)
*T*_min_, *T*_max_	0.791, 1.000
No. of measured, independent and observed [*I* > 2σ(*I*)] reflections	5274, 2211, 2042
*R*_int_	0.020
(sin θ/λ)_max_ (Å^−1^)	0.639

Refinement	
*R*[*F* ^2^ > 2σ(*F* ^2^)], *wR*(*F* ^2^), *S*	0.043, 0.125, 1.09
No. of reflections	2211
No. of parameters	165
H-atom treatment	H-atom parameters constrained
Δρ_max_, Δρ_min_ (e Å^−3^)	0.37, −0.30

Computer programs: *CrysAlis PRO* (Rigaku OD, 2022 ▸), *SHELXT* (Sheldrick, 2015*a* ▸), *SHELXL* (Sheldrick, 2015*b* ▸), *DIAMOND* (Brandenburg & Putz, 1999 ▸), *XP* in *SHELXTL-PC* (Sheldrick, 2008 ▸) and *publCIF* (Westrip, 2010 ▸).

## supplementary crystallographic information

### Crystal data

**Table:**

C_22_H_18_N_2_O_12_	*Z* = 1
*M**_r_* = 502.38	*F*(000) = 260
Triclinic, *P*1	*D*_x_ = 1.561 Mg m^−^^3^
*a* = 5.9454 (2) Å	Cu *K*α radiation, λ = 1.54184 Å
*b* = 7.9543 (3) Å	Cell parameters from 3255 reflections
*c* = 12.0673 (4) Å	θ = 3.9–78.8°
α = 72.124 (3)°	µ = 1.12 mm^−^^1^
β = 79.661 (3)°	*T* = 100 K
γ = 86.052 (3)°	Plate, colourless
*V* = 534.25 (3) Å^3^	0.19 × 0.08 × 0.02 mm

### Data collection

**Table:**

XtaLAB Synergy, Dualflex, HyPix diffractometer	2211 independent reflections
Radiation source: micro-focus sealed X-ray tube, PhotonJet (Cu) X-ray Source	2042 reflections with *I* > 2σ(*I*)
Mirror monochromator	*R*_int_ = 0.020
Detector resolution: 10.0000 pixels mm^-1^	θ_max_ = 80.1°, θ_min_ = 3.9°
ω scans	*h* = −7→7
Absorption correction: multi-scan (CrysAlisPro; Rigaku OD, 2022)	*k* = −9→6
*T*_min_ = 0.791, *T*_max_ = 1.000	*l* = −15→15
5274 measured reflections	

### Refinement

**Table:**

Refinement on *F*^2^	Primary atom site location: dual
Least-squares matrix: full	Hydrogen site location: inferred from neighbouring sites
*R*[*F*^2^ > 2σ(*F*^2^)] = 0.043	H-atom parameters constrained
*wR*(*F*^2^) = 0.125	*w* = 1/[σ^2^(*F*_o_^2^) + (0.071*P*)^2^ + 0.2093*P*] where *P* = (*F*_o_^2^ + 2*F*_c_^2^)/3
*S* = 1.09	(Δ/σ)_max_ < 0.001
2211 reflections	Δρ_max_ = 0.37 e Å^−^^3^
165 parameters	Δρ_min_ = −0.30 e Å^−^^3^
0 restraints	

### Special details

**Table:**

Geometry. All esds (except the esd in the dihedral angle between two l.s. planes) are estimated using the full covariance matrix. The cell esds are taken into account individually in the estimation of esds in distances, angles and torsion angles; correlations between esds in cell parameters are only used when they are defined by crystal symmetry. An approximate (isotropic) treatment of cell esds is used for estimating esds involving l.s. planes.

### Fractional atomic coordinates and isotropic or equivalent isotropic displacement parameters (Å^2^)

**Table:**

	*x*	*y*	*z*	*U*_iso_*/*U*_eq_	
C1	0.0359 (3)	0.5721 (2)	0.51241 (15)	0.0281 (3)	
H1	−0.031345	0.684945	0.481716	0.034*	
C2	0.2167 (3)	0.55372 (19)	0.58634 (13)	0.0233 (3)	
C3	0.3159 (3)	0.69811 (19)	0.60246 (13)	0.0227 (3)	
C4	0.4711 (3)	0.6807 (2)	0.67872 (13)	0.0231 (3)	
H4	0.530524	0.782718	0.687774	0.028*	
C5	0.5389 (3)	0.5136 (2)	0.74157 (13)	0.0228 (3)	
C6	0.4506 (3)	0.36589 (19)	0.72551 (13)	0.0232 (3)	
C7	0.2901 (3)	0.3868 (2)	0.65094 (14)	0.0240 (3)	
H7	0.228245	0.284508	0.643620	0.029*	
N1	0.2610 (2)	0.88106 (17)	0.53758 (12)	0.0251 (3)	
O1	0.2657 (2)	0.99488 (15)	0.58698 (11)	0.0322 (3)	
O2	0.2220 (2)	0.91252 (15)	0.43721 (11)	0.0326 (3)	
C8	0.6966 (3)	0.50015 (19)	0.82811 (13)	0.0232 (3)	
O3	0.8602 (2)	0.59305 (14)	0.80816 (10)	0.0283 (3)	
O4	0.62797 (19)	0.37663 (14)	0.93005 (9)	0.0258 (3)	
C9	0.7826 (3)	0.3411 (2)	1.01503 (15)	0.0302 (4)	
H9A	0.933156	0.306373	0.979732	0.045*	
H9B	0.722258	0.245232	1.085262	0.045*	
H9C	0.796944	0.447755	1.037561	0.045*	
C10	0.5267 (3)	0.1811 (2)	0.78464 (14)	0.0259 (3)	
O5	0.3990 (2)	0.05833 (16)	0.82506 (13)	0.0407 (3)	
O6	0.75063 (19)	0.17046 (14)	0.78393 (10)	0.0261 (3)	
C11	0.8374 (3)	−0.0025 (2)	0.84384 (17)	0.0332 (4)	
H11A	0.806798	−0.087882	0.804802	0.050*	
H11B	0.761355	−0.039755	0.926402	0.050*	
H11C	1.002594	0.003374	0.840751	0.050*	

### Atomic displacement parameters (Å^2^)

**Table:**

	*U*^11^	*U*^22^	*U*^33^	*U*^12^	*U*^13^	*U*^23^
C1	0.0292 (8)	0.0238 (8)	0.0394 (9)	0.0083 (6)	−0.0167 (7)	−0.0170 (6)
C2	0.0255 (7)	0.0214 (7)	0.0276 (7)	0.0048 (5)	−0.0089 (6)	−0.0128 (6)
C3	0.0273 (7)	0.0172 (7)	0.0268 (7)	0.0060 (5)	−0.0088 (6)	−0.0099 (5)
C4	0.0268 (7)	0.0186 (7)	0.0279 (7)	0.0021 (5)	−0.0088 (6)	−0.0110 (6)
C5	0.0243 (7)	0.0207 (7)	0.0264 (7)	0.0035 (5)	−0.0081 (6)	−0.0100 (6)
C6	0.0255 (7)	0.0188 (7)	0.0276 (7)	0.0033 (5)	−0.0082 (6)	−0.0093 (6)
C7	0.0268 (7)	0.0187 (7)	0.0316 (8)	0.0030 (5)	−0.0105 (6)	−0.0124 (6)
N1	0.0276 (6)	0.0196 (6)	0.0319 (7)	0.0046 (5)	−0.0117 (5)	−0.0107 (5)
O1	0.0396 (7)	0.0196 (6)	0.0455 (7)	0.0080 (4)	−0.0185 (5)	−0.0169 (5)
O2	0.0418 (7)	0.0262 (6)	0.0326 (6)	0.0038 (5)	−0.0183 (5)	−0.0071 (5)
C8	0.0282 (7)	0.0166 (7)	0.0289 (7)	0.0053 (5)	−0.0105 (6)	−0.0110 (6)
O3	0.0309 (6)	0.0226 (6)	0.0356 (6)	0.0005 (4)	−0.0138 (5)	−0.0102 (4)
O4	0.0327 (6)	0.0202 (5)	0.0272 (5)	0.0016 (4)	−0.0126 (4)	−0.0069 (4)
C9	0.0366 (8)	0.0265 (8)	0.0311 (8)	0.0045 (6)	−0.0171 (7)	−0.0085 (6)
C10	0.0306 (8)	0.0193 (7)	0.0319 (8)	0.0021 (6)	−0.0137 (6)	−0.0094 (6)
O5	0.0386 (7)	0.0223 (6)	0.0605 (8)	−0.0023 (5)	−0.0246 (6)	−0.0018 (5)
O6	0.0297 (6)	0.0185 (5)	0.0333 (6)	0.0066 (4)	−0.0129 (4)	−0.0097 (4)
C11	0.0373 (9)	0.0200 (8)	0.0443 (9)	0.0099 (6)	−0.0173 (7)	−0.0084 (7)

### Geometric parameters (Å, º)

**Table:**

C1—C1^i^	1.383 (3)	N1—O1	1.2316 (17)
C1—H1	0.9500	N1—O2	1.2208 (18)
C1—C2	1.488 (2)	C8—O3	1.2065 (19)
C2—C3	1.406 (2)	C8—O4	1.3335 (19)
C2—C7	1.403 (2)	O4—C9	1.4497 (18)
C3—C4	1.388 (2)	C9—H9A	0.9800
C3—N1	1.4710 (19)	C9—H9B	0.9800
C4—H4	0.9500	C9—H9C	0.9800
C4—C5	1.386 (2)	C10—O5	1.202 (2)
C5—C6	1.398 (2)	C10—O6	1.3268 (19)
C5—C8	1.5000 (19)	O6—C11	1.4515 (18)
C6—C7	1.394 (2)	C11—H11A	0.9800
C6—C10	1.502 (2)	C11—H11B	0.9800
C7—H7	0.9500	C11—H11C	0.9800
			
C1^i^—C1—H1	119.4	O2—N1—C3	118.67 (12)
C1^i^—C1—C2	121.20 (18)	O2—N1—O1	123.86 (13)
C2—C1—H1	119.4	O3—C8—C5	123.87 (14)
C3—C2—C1	123.55 (13)	O3—C8—O4	124.94 (14)
C7—C2—C1	121.09 (13)	O4—C8—C5	111.17 (13)
C7—C2—C3	115.26 (13)	C8—O4—C9	115.16 (12)
C2—C3—N1	121.38 (13)	O4—C9—H9A	109.5
C4—C3—C2	123.52 (13)	O4—C9—H9B	109.5
C4—C3—N1	115.10 (12)	O4—C9—H9C	109.5
C3—C4—H4	120.2	H9A—C9—H9B	109.5
C5—C4—C3	119.57 (13)	H9A—C9—H9C	109.5
C5—C4—H4	120.2	H9B—C9—H9C	109.5
C4—C5—C6	118.99 (13)	O5—C10—C6	123.44 (14)
C4—C5—C8	117.98 (13)	O5—C10—O6	124.79 (14)
C6—C5—C8	122.98 (13)	O6—C10—C6	111.75 (13)
C5—C6—C10	122.09 (13)	C10—O6—C11	115.35 (12)
C7—C6—C5	120.29 (13)	O6—C11—H11A	109.5
C7—C6—C10	117.60 (13)	O6—C11—H11B	109.5
C2—C7—H7	118.9	O6—C11—H11C	109.5
C6—C7—C2	122.30 (13)	H11A—C11—H11B	109.5
C6—C7—H7	118.9	H11A—C11—H11C	109.5
O1—N1—C3	117.41 (12)	H11B—C11—H11C	109.5
			
C1^i^—C1—C2—C7	−11.9 (3)	C1—C2—C7—C6	−176.69 (14)
C1^i^—C1—C2—C3	171.9 (2)	C4—C3—N1—O2	146.08 (15)
C7—C2—C3—C4	−1.7 (2)	C2—C3—N1—O2	−33.6 (2)
C1—C2—C3—C4	174.69 (15)	C4—C3—N1—O1	−31.3 (2)
C7—C2—C3—N1	177.99 (13)	C2—C3—N1—O1	148.99 (15)
C1—C2—C3—N1	−5.6 (2)	C4—C5—C8—O3	−43.2 (2)
C2—C3—C4—C5	1.5 (2)	C6—C5—C8—O3	139.53 (16)
N1—C3—C4—C5	−178.24 (13)	C4—C5—C8—O4	134.90 (14)
C3—C4—C5—C6	0.7 (2)	C6—C5—C8—O4	−42.34 (19)
C3—C4—C5—C8	−176.63 (13)	O3—C8—O4—C9	−8.0 (2)
C4—C5—C6—C7	−2.5 (2)	C5—C8—O4—C9	173.88 (12)
C8—C5—C6—C7	174.67 (14)	C7—C6—C10—O5	−40.6 (2)
C4—C5—C6—C10	176.16 (14)	C5—C6—C10—O5	140.64 (18)
C8—C5—C6—C10	−6.6 (2)	C7—C6—C10—O6	137.89 (15)
C5—C6—C7—C2	2.3 (2)	C5—C6—C10—O6	−40.8 (2)
C10—C6—C7—C2	−176.45 (14)	O5—C10—O6—C11	−4.0 (2)
C3—C2—C7—C6	−0.2 (2)	C6—C10—O6—C11	177.52 (13)

Symmetry code: (i) −*x*, −*y*+1, −*z*+1.

### Hydrogen-bond geometry (Å, º)

**Table:**

*D*—H···*A*	*D*—H	H···*A*	*D*···*A*	*D*—H···*A*
C9—H9*B*···O5^ii^	0.98	2.42	3.308 (2)	150
C11—H11*A*···O3^iii^	0.98	2.52	3.3650 (19)	144
C11—H11*C*···O5^iv^	0.98	2.39	3.364 (2)	173

Symmetry codes: (ii) −*x*+1, −*y*, −*z*+2; (iii) *x*, *y*−1, *z*; (iv) *x*+1, *y*, *z*.
